# Failure to Thrive: Impaired BDNF Transport along the Cortical–Striatal Axis in Mouse Q140 Neurons of Huntington’s Disease

**DOI:** 10.3390/biology12020157

**Published:** 2023-01-19

**Authors:** Michael T. Maloney, Wei Wang, Sumana Bhowmick, Ivan Millan, Mridu Kapur, Nicolas Herrera, Everett Frost, Elena Y. Zhang, Scott Song, Melissa Wang, Amelia Bora Park, Annabelle Y. Yao, Yanmin Yang

**Affiliations:** Department of Neurology and Neurological Sciences, Stanford University School of Medicine, 1201 Welch Road, MSLS, P259, Stanford, CA 94305, USA

**Keywords:** Huntington’s disease, Q140 mice, brain-derived neurotrophic factor, medium spiny neuron, cortical–striatal axis

## Abstract

**Simple Summary:**

The brain-derived neurotrophic factor (BDNF) is implicated in the function and survival of striatal neurons. Deficits in neurotrophic support by BDNF have been hypothesized to contribute to the neurodegeneration of striatal neurons in Huntington’s disease (HD). This article outlines an investigation into defects in the transport of BDNF along the cortical–striatal axis in primary neurons isolated from the Q140 mouse model of HD. Herein we employ a microfluidic chamber cell culture system specifically designed to grow cortical–striatal co-cultures. Within this system, we employed quantum dot-conjugated BDNF (QD-BDNF) to visualize single-molecule transport behaviors of BDNF undergoing axonal and dendritic transport. We found a global defect in the supply of QD-BDNF moving anterogradely along cortical axons, and retrogradely within the striatal dendrites of Q140 primary neurons. These findings support the notion that the essential spatiotemporal support from BDNF to striatal neurons is reduced due to deficits in its cortical–striatal transport, a defect contributing to striatal neurodegeneration in HD. BDNF trophic support has been proposed as a therapeutic intervention to delay or prevent the onset and progression of neurodegenerative diseases. Our study provides additional insight into how therapeutic strategies involving BDNF supplementation may be applied for Huntington’s or other diseases.

**Abstract:**

Boosting trophic support to striatal neurons by increasing levels of brain-derived neurotrophic factor (BDNF) has been considered as a target for therapeutic intervention for several neurodegenerative diseases, including Huntington’s disease (HD). To aid in the implementation of such a strategy, a thorough understanding of BDNF cortical–striatal transport is critical to help guide its strategic delivery. In this manuscript, we investigate the dynamic behavior of BDNF transport along the cortical–striatal axis in Q140 primary neurons, a mouse model for HD. We examine this by using single-molecule labeling of BDNF conjugated with quantum dots (QD-BDNF) to follow the transport along the cortical–striatal axis in a microfluidic chamber system specifically designed for the co-culture of cortical and striatal primary neurons. Using this approach, we observe a defect of QD-BDNF transport in Q140 neurons. Our study demonstrates that QD-BDNF transport along the cortical–striatal axis involves the impairment of anterograde transport within axons of cortical neurons, and of retrograde transport within dendrites of striatal neurons. One prominent feature we observe is the extended pause time of QD-BDNF retrograde transport within Q140 striatal dendrites. Taken together, these finding support the hypothesis that delinquent spatiotemporal trophic support of BDNF to striatal neurons, driven by impaired transport, may contribute to the pathogenesis of HD, providing us with insight into how a BDNF supplementation therapeutic strategy may best be applied for HD.

## 1. Introduction

Huntington’s disease (HD) is a progressive, dominantly inherited neurodegenerative disorder which is estimated to affect approximately one in every 10,000 people, with nearly 30,000 patients in the United States [1]. It is caused by the expansion of polyglutamine repeat (polyQ), encoded by the codon CAG, within the huntingtin (*HTT*) gene. HD occurs spontaneously and is subsequently transmitted through generations with a concomitant increase in CAG/polyglutamine repeat number, a process described as genetic anticipation [2]. The symptoms of HD vary between individuals but are usually characterized by a triad of motor, cognitive, and psychiatric manifestations [3]. Neuropathologically, HD is marked by loss of specific neurons in the brain. Loss of neurons in the striatum and its effect on the motor neurons projecting from cortex have been particularly reported [4]. Huntingtin (HTT), a 3144 amino acid protein, traced back over millions of years, shuttles between the nucleus and the cytoplasm via active transport regulating its aggregation, subcellular localization, and toxicity [5]. HTT is involved in neural tube formation, neuroblast migration, axonal transport, synaptic function and cell survival [4,6]. Mutation in HTT (mHTT) results in abnormal expansion of CAG repeats (exceeding 36 repeats), conferring an increased risk for the development of HD [7]. Alongside alterations of the central nervous system (CNS), the most prominent clinical features of HD, patients also suffer from metabolic and immune disturbances, skeletal-muscular wasting, weight loss, cardiac failure, testicular atrophy, and osteoporosis [4]. 

Mammalian neurotrophins (NTs) regulate cellular differentiation, survival, maintenance, and synaptic plasticity in the peripheral and central nervous systems [8]. Among all the NTs, BDNF is the most widely distributed and extensively studied as it plays pivotal roles in the neuronal function of the peripheral and central nervous systems. BDNF’s role is to particularly regulate numerous physiological brain processes based on its interaction with different types of receptors [9]. BDNF binds with high affinity to the tropomyosin-receptor-kinase B (TrkB) receptor, which in turn activates several small G-protein kinases as well as pathways regulated by phosphatidylinositol 3-kinase (PI3K)/protein kinase B (Akt), MAP kinase-interacting kinases (MNK), and the mechanistic target of rapamycin (mTOR) signaling [10,11,12]. Interest in increasing BNDF expression or otherwise boosting levels of BDNF in the brain as a strategy to prevent neurodegeneration has grown in popularity. This is in large part because of its key role in supporting the function, maturation and survival of substantia nigra (SN) and nigral dopaminergic (DA) neurons, as well as its influence on the structure and function of striatal medium spiny neurons (MSNs) [13,14,15]. Furthermore, other studies have reported a vital role for BDNF in aiding the establishment of striatal connections during brain development, actin remodeling of MSNs, and dendritic spine dynamics [16]. 

HTT has been reported to promote BDNF expression in the cytoplasm and to enhance BDNF vesicular trafficking along microtubules [17]. Thus, mHTT may interfere with these functions, along with reduced BDNF trafficking and a subsequent decrease in striatal BDNF supply [18,19,20,21,22,23,24,25]. While mHTT is the root cause of HD, a clear understanding of mechanisms precipitating deficient BDNF expression and delivery in HD remains incomplete [26,27]. Retrograde fast axonal transport is a vital part of the neuronal system for the conveyance of organelles and endosomal cargoes over long distances [28,29]. Reports of axonal transport disruption participating in neurodegenerative cascades [30,31,32] have been thoroughly investigated. However, for technical reasons, dendritic transport has been difficult to accurately quantify [33,34]. The use of protein overexpression has been widely used to examine transport [35,36,37]; however, these systems produce unavoidable artifacts that corrupt the endogenous nature of the neurite. The microfluidic chamber offers multiple advantages over other systems, the most important of which are: (1) ability to fluidically isolate distal dendrites from cell bodies; and (2) ability when coupled with exogenously applied ligands to allow for the observation of single-molecule transport in a physiologically relevant receptor/ligand setting. The separation of dendrites from neuronal soma using microfluidic devices has proven challenging but has been met with some success [38,39,40]. 

As a vast array of brain functions rely on BDNF-TrkB signaling, we initially sought to evaluate the induction of trophic signaling in Q140 neurons when stimulated by BDNF, as has been previously reported in literature. To further understand and explore the specific dysfunctions driving the deficiency of BDNF transport along the cortical–striatal axis in Q140 mice, our study incorporates advances in single-molecule imaging coupled with modified microfluidic primary neuron co-culture chambers. This allows us to carefully examine the complete transport cycle of QD-BDNF along the entire cortical–striatal axis. Described herein, results from this study reveal a selective neurotrophic transport defect in primary cortical axons from the Q140 mouse. In addition, we provide a detailed study of real-time anterograde and retrograde cortical–striatal transport using exogenously applied ligands in a microfluidic platform. Thus, we elucidate details of mechanisms that hamper transport and ultimately reduce the supply of BDNF along the HD cortical axons and striatal dendrites. Our study highlights that the use of single-molecule QD-BDNF can be used to follow axonal and dendritic transport in live cells and build support for a current global model of neurodegeneration. This study supports the utility of this fundamental culture system, which can be easily adapted to evaluate potential therapeutics or enhance the application of therapeutic BDNF resupply strategies for Huntington’s disease or other neurodegenerative diseases.

## 2. Materials and Methods

### 2.1. Animals

All procedures were conducted in accordance with the NIH Guidelines for the Care and Use of Laboratory Animals and protocols were approved by the Institutional Animal Care and Use Committee at Stanford. The knock-in (KI) mice contained a chimeric mouse/human exon 1, with 140 CAG repeats (Q140) inserted into the mouse gene by homologous targeting [41]. All KI mice (>10 pairs) used in this study were homozygous for the mutation, and wild-type (WT) littermates were used as controls. 

### 2.2. Cell Culture and Treatments

MSN-enriched primary striatal neurons were dissociated from mouse embryos (E14–16) of WT C57BL/6 and HTT Q140 mice, as described previously with the following changes [42,43,44]. Dissociated cells were plated on 100 µg/mL poly-L-lysine-coated substrates. For live imaging, neurons were plated into microfluidic chambers affixed to 22 × 30 mm cover glass in microfluidic chambers. Neurons were plated in Plating Medium (Neurobasal media, 10% FBS, antibiotic/antimycotic) (Invitrogen, Waltham, MA, USA). After 3 h, the medium was changed to a serum-free formula Neurobasal/B27 (Neurobasal, B27 supplement, GlutaMax) (Invitrogen). One half of the medium was changed every 2 days and replaced with fresh medium. Neurons were cultured in a humidified incubator at 36 °C, 95% air and 5% CO_2_. HEK-293-T, 3T3 stable expressing TrkA or TrkB cells were cultured in DMEM medium (Invitrogen), supplemented with 10% FBS and cultured in a humidified incubator at 37 °C, 95% air and 5% CO_2_. 

### 2.3. btBDNF Cloning

Pre-pro-BDNF was amplified by PCR from the mouse brain cDNA library using reverse transcriptase. PCR was used to add the 17 amino acid AviTag sequence: GGGLNDIFEAQKIEWHE. It was based on the #85 AviTag peptide sequence described previously [45]. In addition, one glutamic acid residue was added to the C-terminal AviTag vectors to enhance the biotinylation rates of the AviTag 14 (Avidity, Aurora, CO, USA) [46]. The PCR product was purified and digested with EcoRI and BamHI and then ligated into the pcDNA3.1-myc-His vector, designated as pcDNA3.1-BDNFavi. BirA was amplified by PCR from pET2-BirA (Addgene, Watertown, MA, USA) [47]. The PCR product was digested with XhoI and was sub-cloned into a pcDNA3.1-myc-his (+) vector (Invitrogen) restricted with EcoRV and XhoI. The resulting plasmid was designated as pcDNA3.1-BirA. All primers were purchased from Elim Biopharmaceuticals, Inc., Hayward, CA, USA. All constructs were verified by sequencing.

### 2.4. Biotinylated Mature BDNF (btBDNF) Production

To produce btBDNF, HEK-293-T cells were grown on 150 mm plates to ~70% confluence. Cells were changed to DMEM serum-free media, supplemented with 50µM D- (+) -Biotin (Sigma-Aldrich, Burlington, MA, USA) and were transfected with 20µg pcDNA3.1-BDNFavi plasmid DNA and 20µg pcDNA3.1-BirA plasmid DNA using Turbofect (Thermo Fisher Scientific, Waltham, MA, USA) following the manufacture’s instruction. Media were collected for protein purification 72 h post transfection.

### 2.5. btBDNF Protein Purification

The media containing btBDNF were harvested and plates were washed using a washing buffer (30 mM phosphate buffer (pH 8.0), 500 mM NaCl, 20 mM imidazole and a cocktail of protease inhibitors) (Sigma-Aldrich). The media were then incubated on ice for 15 min, centrifuged at 18,000 rpm for 30 min using a Beckman JA-20 rotor and the supernatant was collected. Ni-NTA resins (Qiagen, Hilden, Germany) were rinsed with the washing buffer and added to the collected supernatant at a concentration of 0.3 mL Ni-NTA resins to 100 mL media and incubated overnight on a shaker at 4 °C. The media/Ni-NTA slurry was loaded onto a column and the captured Ni-NTA resins were washed with 10 mL wash buffer and eluted with the elution buffer (30 mM phosphate buffer, pH 8.0, 500 mM NaCl, 300 mM imidazole, protease inhibitors). The purity and concentration of BDNF was assessed by SDS-PAGE using a fast silver staining kit (G-Biosciences, St Louis, MO, USA). Known quantities of BDNF and BSA were used as standards. To precipitate proteins with trichloroacetic acid (TCA), 100% ice-cold TCA was added to the supernatant at a final concentration of 5–7% and incubated on ice for 20 min. The sample was then centrifuged for 20 min at 14,000 rpm. The pellet was washed three times with acetone, air-dried and heated in an SDS loading buffer.

### 2.6. Microfluidic Chambers

Microfluidic chambers used in these experiments are designed in our lab and manufactured at the Stanford Nanofabrication Facility, essentially as described previously [48]. The chamber design allows for cell bodies to be grown in one compartment while axons and dendrites are directed to another adjacent compartment through embedded microgrooves. In addition, fluidic isolation can be created between the cell body and axonal compartments by maintaining a slightly higher liquid level in the cell body compartment. This prevents the diffusion of analytes from the distal axon compartment into the cell body compartment. Briefly, blueprints produced using computer aided design software (L Edit v12, Tanner EDA Software tools) were used to make high-resolution 20,000 dpi. transparency masks for photolithography. Masks were used to pattern 100 mm silicon wafers for master templates beginning with SU8-2005 to create 5 µm features (microgrooves), followed by SU8-2050 for 100 µm features (cell body and distal axon chambers). Finally, polydimethylsiloxane (Sylgard 184 Elastomer; Dow Corning, Midland, MI, USA) molds, produced using the master template, were cut to final dimensions, and used as microfluidic chambers. Microfluidic chambers were placed onto poly-D-lysine-coated coverslips for neuronal culture. 

### 2.7. Live Imaging of QD Transport

Dissociated neurons were plated into the cell body chamber of a microfluidic device. Axons or dendrites started to cross the first set of microgrooves into the middle chamber within ~5–7 days. To prepare the QD-BDNF, 100 nM of btBDNF dimer was mixed with 100 nM QD625-streptavidin conjugates (Invitrogen) and incubated overnight at 4 °C. BDNF-QD625 was diluted to a final concentration of 0.2 nM in Neurobasal media *w/o* phenol red (Invitrogen) and added to the cortical soma body chamber for the axonal anterograde transport study and to the middle chamber for the striatal dendrite’s retrograde transport study. After 30 min incubation at 37 °C, live cell imaging of the QD625 transport was carried out using a Leica DMI6000B inverted microscope. The scope was equipped with an environmental chamber that maintained a constant conditions of 37 °C and 5% CO_2_ throughout imaging. A Texas red excitation/emission filter cube was used to visualize the QD625 signal. Time-lapse images were acquired at the speed of 1 frame/second and were captured using a CCD camera (Rolera-Mgi Fast 1397 from Qimaging). All data were processed and analyzed using ImageJ. We took 2–4 min movies, with a frame acquired every 1 s. QDs were tracked using the Kymograph and MtrackJ feature in ImageJ. On average, 10–20 QDs were tracked per microfluidic chamber. Post-analysis was performed in Microsoft Excel. A paused quantum dot was defined as one that was moving less than 0.5 µm/s in a non-processive manner. Average speed was calculated based on the final displacement of the quantum dot. The active moving velocity only included quantum dots during periods of active movement and ignored times in which a quantum dot was paused. Statistical analysis was performed in Excel or Prism v76.0. 

### 2.8. Antibodies

All the antibodies used are listed in Table 1.

### 2.9. Immunoblot

Treated cells were collected from the P60 dish and centrifuged to remove the media. The cell pellet was suspended in 4 × protein sample loading buffer (Li Cor Biosciences, Lincoln, NE, USA). The samples were loaded into 10% SDS-PAGE gel. The gel was then transferred to PVDF membranes and incubated with blocking buffer (Li Cor) for an hour. The membrane was then incubated with primary antibodies (TuJ1, TrkB, phospho-TrkB, Akt, Erk and phospho-Akt, phospho-Erk, CREB, phospho-CREB, and HTT, Table 1) overnight at 4 °C. The membrane was washed and incubated with HRP-conjugated goat anti-rabbit or mouse second antibody and further with WesternBright™ Quantum (K-12042-D10, Advansta, San Jose, CA, USA). Exposed X-ray film was developed in a Kodak developer machine. β-actin was used as the loading control. All experiments were triplicated for verifications.

### 2.10. Immunostaining

Cells were seeded in 18 mm coverslips and fixed for 12 min in 4% paraformaldehyde with 5% sucrose at pH 7.0. Cells were permeabilized and blocked (3% BSA, 0.1% gelatin, 0.1% Triton-X-100 in TBS pH 7.4) for an hour at room temperature before immunostaining was performed with various antibodies. The primary antibodies (all diluted in blocking buffer) used in immunostaining were: TrkB, TuJ1, MAP2, CREB, phospho-CREB and DARPP-32 (Table 1). This was followed by secondary antibody incubation and DAPI-Fluoromount-G^®^ (0100-20; Southern Biotech, Birmingham, AL, USA) staining. All experiments were repeated in triplicate, with similar results obtained.

### 2.11. Microscopy

All images were captured on a Leica fluorescent inverted microscope DMI6000B. A Qimaging Retiga Exi Fast 1394 camera was used. Movies and images were prepared for presentation in ImageJ or Adobe Illustrator. 

## 3. Results

### 3.1. Effect of BDNF on the BDNF/TrkB Signaling Pathway in Q140 Striatal Neurons

BDNF signaling is considered to be a therapeutic target in HD, especially its role in neuronal survival, signaling and transport. The binding of BDNF to the TrkB receptor is known to promote two key downstream signaling pathways, namely, the mitogen-activated protein kinase (MAPK)/extracellular regulatory protein kinase (Erk) and PI3K/Akt signaling pathways that induces cAMP-response element binding protein (CREB) phosphorylation and activation [14,15,49,50].

**Figure 1:**
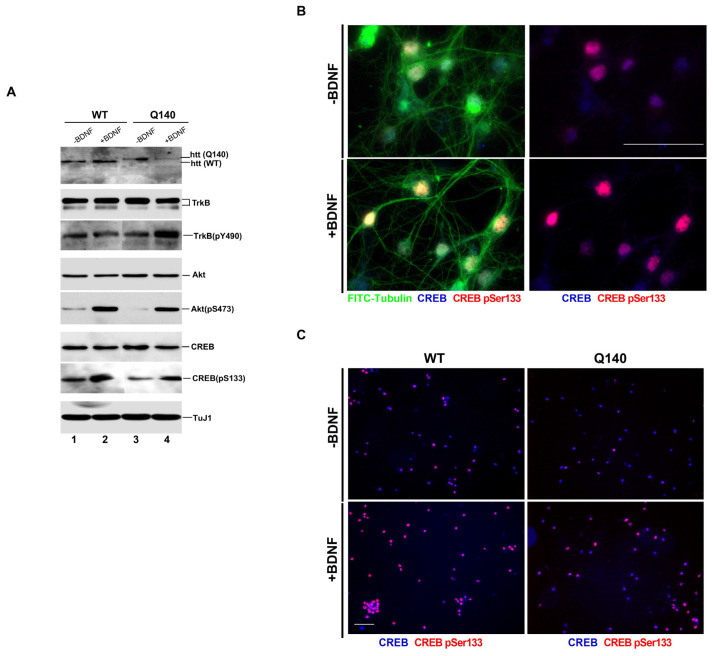
BDNF effect on the BDNF-TrkB signalling pathway in Q140 striatal neurons. (**A**). Representative immunoblots of TuJ1, TrkB, TrkB (pY490), Akt, pAkt (pS473), CREB and pCREB (pS133), and in WT and Q140 cells with and without BDNF (25 ng/mL) for 30 min. We detect reduced levels of pAkt (p8473) and pCREB (pS133) in Q140 (lane 3 compared to lane 1 in WT. Repeated in triplicates. (**B**). Striatal neuron staining with FITC-tubulin CREB and pCREB (pSer133) confirms that the activation of CREB into pCREB in WT occurs with BDNF after 30 min. (**C**). Staining with CREB and pCREB (pSer133) shows alterations of pCREB in WT and Q140 after 30 min inductions, with or without BDNF (25 ng/mL). Repeated in triplicates. Scale bar is 100 μm.

To confirm BDNF’s effects on downstream signaling pathways, including key signaling molecules TrkB, Akt, Erk and CREB, immunostaining and immunoblotting were conducted on WT and Q140 striatal neurons (Figure 1). The results in Figure 1A showed that, without BDNF induction, a diminished level of active forms of Akt (pAkt) and CREB (pCREB) in Q140 samples (lane 3 in comparison with lane 1), while no significant change was observed in the total levels of TrkB, Akt, and CREB (lane 3 in comparison with lane 1). We next induced the WT and Q140 neurons with BDNF for 30 min to monitor their alterations. Our data shows that treatment with BDNF can boost the levels of pAkt and pCREB in Q140 neurons to a quite comparable level in WT neurons (lane 2 and lane 4), implying that the lower levels of these downstream signaling molecules in untreated Q140 samples could be a result of deficiency in proper supply of BDNF. However, the 30 min treatment with BDNF had no alteration in pTrkB levels, consistent with other studies which showed that the pTrkB level may drop down at 30 min in response to BDNF being induced in cortical neurons [51,52,53]. The differences of pCREB, with or without BDNF induction, can be visualized by immunostaining (Figure 1B and 1C). Our results are consistent with other studies published on the effects of BDNF on downstream signaling molecules in HD [20,54]. Surprisingly, the pTrkB level in Q140 striatal neuron was observed to be higher compared to the WT. One possible reason for this could be the defect of BDNF-TrkB signaling endosomes from synapse to the soma [55]. Taking into account previous study on decreases in BDNF release and the total travel and speed of BDNF vesicles in cortical neurons [16], we speculate that neurodegeneration in HD may attribute to the lack of spatiotemporal supplies of BDNF along the cortical–striatal axis in neurons.

### 3.2. QD625-Streptavidin Is Conjugated with BDNF 

To visualize the dynamic behavior of BDNF, the QD-BDNF was designed experimentally for our study (Figure S1). To conjugate with QD625-streptavidin, BDNF was required to be biotinylated, as previously described in several studies [52,56,57]. The biotin-BDNF and BirA plasmids were constructed for expression in the mammalian system. His tag-incorporated btBDNF was then purified with the Ni-NTA resins from transfected cell culture medium and was finally eluted with BDNF-Avi (biotin)-His (Biotin-BDNF, btBDNF), showing a band of 18 KDa (Figure S2A). Further, the band was confirmed with His (data not shown), BDNF and Biotin antibodies (Figure S2B). Measurement of the concentration of purified btBDNF was determined by comparison against known concentrations of recombinant BDNF and determined to be ~2 ng/µL (Figure S2C). To further assess the biological activity of btBDNF, we used TrkB-expressing and TrkA-expressing 3T3 cell lines. It has been previously reported that BDNF binding to its cognate receptor TrkB leads to the phosphorylation of downstream substrates, including Akt and Erk 42/44 [58]. As expected, Akt and Erk were activated in TrkB-3T3 cells treated with purified btBDNF, or when treated with purchased purified BDNF (Figure S3A). In addition, endosomal TrkB were also observed (Figure S3B). Similar effects of btBDNF were also observed in the cultured neurons [59]. Finally, we observed live endosomal transport of biologically active btBDNF, conjugated with QD625-streptavidin (QD-BDNF) using fluorescent imaging in the TrkB-expressing 3T3 cells (Figure S3C). This confirms that our purified btBDNF was suitable for use in our neuronal transport assay.

### 3.3. Cortical–Striatal Neuronal Microfluidic Chamber Was Designed and Fabricated for the Study

**Figure 2:**
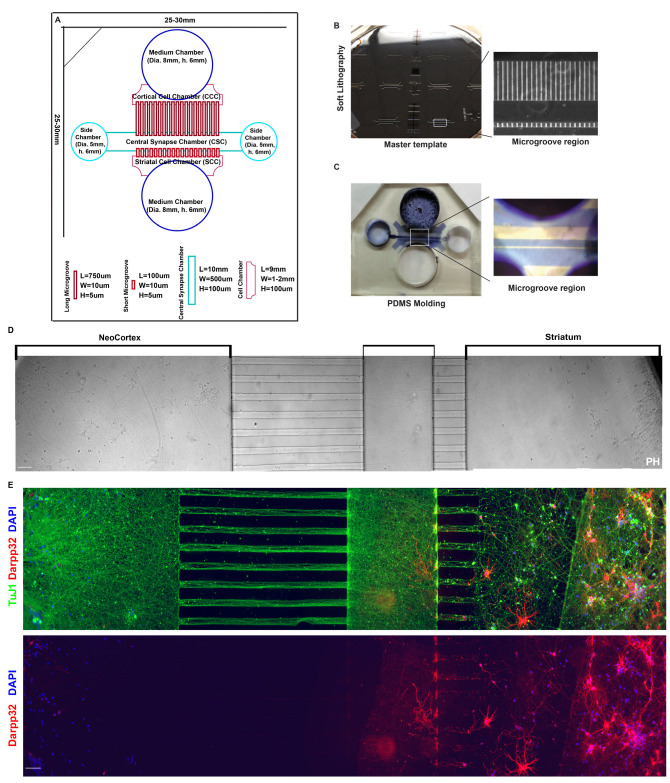
Design and Fabrication of Cortical–Striatal Neuronal Microfluidic Chamber. (**A**). Photograph of the chambers’ AutoCAD design with detailed numbers. (**B**). Photograph of the master template, a 100 mm silicon wafer of crosslinked SU8 negative resists, containing microfluidic channel features. Inset from (**B**) showing detail of long (750 μm, for axon isolation) and short (100 μm, for dendrite isolation) microgrooves separated by a central chamber. (**C**). Low power (5 ×) image of cut PDMS microfluidic chamber. Inset from (**C**) showing cell culture areas in blue and microgrooves in white. Microfluidic chamber with directional cortico-striatal co-culture. (**D**). Differential interference contrast (DIC) to show neurons growing in chamber. (**E**). Further confirmation of neuron in chamber via immunostaining. Microtubule (TuJ1) staining reveals axons growing through the chamber grooves (Top). The DARPP-32 striatal neuron marker confirms the cortex and striatum separation across the left and right chambers (Bottom). Scale bar is 100 μm.

To create a functional cortical–striatal circuit in vitro which can be well mimic the relevant in vivo architecture, the designed chamber featured a dedicated synaptic chamber for cortical axons to synapse onto striatal dendrites [60]. This central chamber was connected to the cortical cell body chamber through 750 µm long microgrooves to allow axons to move into the central chamber. Conversely, the striatal chamber was connected to the central chamber by 100–200 µm long microgrooves to permit dendritic protrusion. All microgrooves were 10 µm in width and 5 µm in height. The new design was produced using a transparency mask and soft lithography to create a master template, from which polydimethylsiloxane (PDMS) molds of the final chamber device were cut (Figure 2A–C). To mimic the in vivo circuit in an in vitro environment, two different populations of neurons from the cortex and striatum can be cultured in the opposing chambers. In Figure 2D, neurons from the neo-cortex and striatum were cultured at different chambers until 8 days (DIV 8), and phase images showed that axons from the neo-cortex passing through the long grooves interacted with dendrites from the striatum passing through the short grooves. Furthermore, the chamber was fixed and confirmed by immunostaining with TuJ1, a neuron-specific beta-tubulin antibody, and DARPP-32, highly recognized as a marker for striatal neurons [61] (Figure 2E). Ultimately, the designed and fabricated microfluidic system proved to be a suitable model for studying the cortical–striatal transport.

### 3.4. Anterograde QD-BDNF Transport Is Hindered in the Q140 Cortical Axon 

Anterograde transport underlies the delivery of neurotrophins, including BDNF and neurotransmitters among other cargoes, from cortical soma to distal axons and presynaptic locations. In HD, it has been hypothesized that the disruption of cortical neuron axonal transport plays a critical role in promoting the degeneration of MSNs in the striatum [18,62,63], though this hypothesis has been challenged. To examine the anterograde component of axonal transport in cortical axons in the context of HD, we cultured neurons from WT and Q140 embryos (E14–16) in the designed co-culture microfluidic chambers. Here, we established fluidic isolation to prevent diffusion into the axon chamber, before adding QD-BDNF to cortical soma in the cell body chamber. After 1 h, axonal transport was examined in the long microgrooves. A distinguished difference between retrograde and anterograde transport was prominently observed. Normally, retrograde transport in WT cortical axons proceeds exclusively in one direction. However, in our condition, QD-BDNF-containing endosomes were observed moving in two directions within the axons, both anterograde and retrograde, with an equal frequency distribution (Figure S4A). Irrespective of their direction of travel, endosomes tended to maintain one direction of travel through the field of view. Upon closer examination, it was observed that occasionally endosomes paused before switching direction (Figure S4B). This observation suggests that, whether anterograde or retrograde in direction, endosomes are governed by a mechanism that maintains one-directional transport over long distances. However, there are reasons behind the reverse direction of an endosome, after which a new direction will again be maintained over a long range, remains unclear.

**Figure 3:**
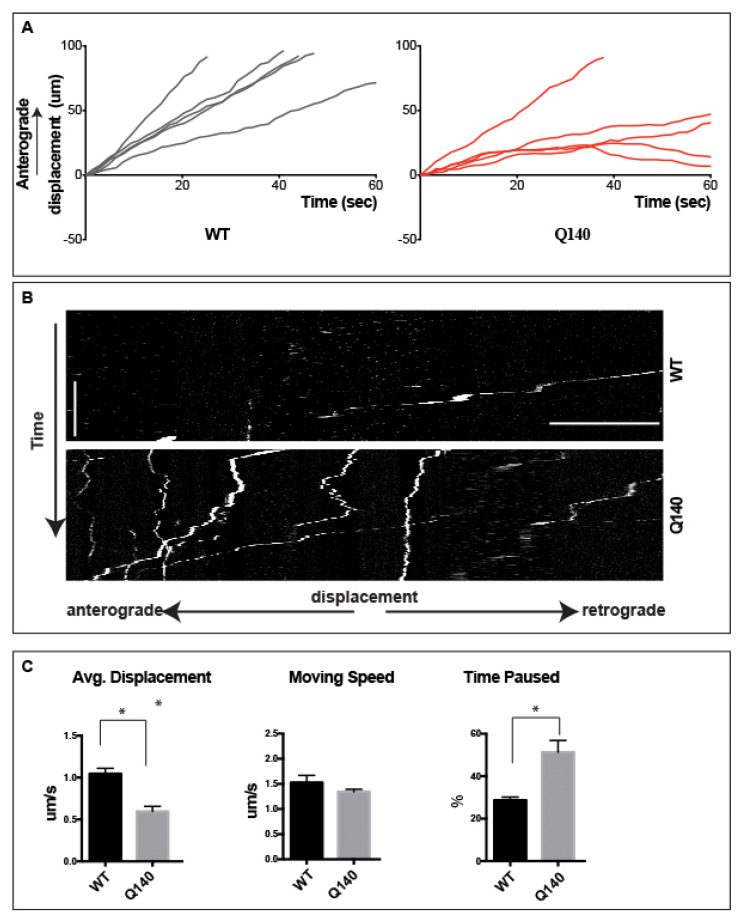
Perturbed anterograde transport of QD-BDNF in Q140 cortical axons. (**A**). Displacement vs. time plots of QD-BDNF anterograde transport in axons of WT (gray) and Q140 (red) cortical neurons. (**B**). Kymographs detailing single-molecule cortical axon transport. (**C**). Bar graphs quantifying averaged displacement, moving speed and percent time paused for QD-BDNF in WT and Q140 cortical axons. Data were from three individual repeats. Vertical Scale bar = 1 min, horizontal scale bar = 5 μm, * *p* < 0.01.

To focus on the anterograde transport profile, the retrograde direction in cortical axons’ endosomes were excluded from analysis. The average anterograde displacement for QD-BDNF in WT cortical axons was 1.05 ± 0.1 µm/s. The average displacement of anterograde QD-BDNF in Q140 cortical axons was significantly reduced as compared to WT (*p* < 0.01; 0.60 ± 0.1 µm/s; Figure 3A). These data demonstrate that there is a reduction of the average displacement of anterograde moving QD-BDNF endosomes within the Q140 cortical axons as compared to WT. As with the motor component, kinesin, we calculated the moving speed of QD-BDNF by measuring the velocity of anterograde endosomes during periods of active transport, eliminating time spent paused. The average moving speed for QD-BDNF was 1.53 ± 0.1 µm/s in WT cortical axons and was reduced to 1.3 ± 0.1 µm/s in Q140 axons, though this did not reach significance (Figure 3B). The finding that moving speeds were unchanged for anterograde endosomes suggests that dysfunction of the kinesin motor is not likely to produce the observed decrease in the average anterograde displacement of endosomes in Q140 cortical axons.

To better understand the mechanism(s) underlying the average displacement data, we next measured the percentage of time spent paused of anterograde endosomes. The average percent time paused for QD-BDNF containing endosomes in WT cortical axons was 39.7 ± 2.5%, while in Q140 cortical axons the average percent time paused for QD-BDNF was significantly increased (*p* < 0.01) to 51.3 ± 5.5% (Figure 3C, Movie 1). From these data, it becomes clear that anterograde transport for BDNF endosomes significantly reduces along with increased pausing in Q140 cortical axons, resulting in axonal transport alterations as compared to WT, and thus underlies the decreased average displacement.

### 3.5. Prolonged pauses dominate the QD-BDNF Transport in MSN Striatal Dendrites 

**Figure 4:**
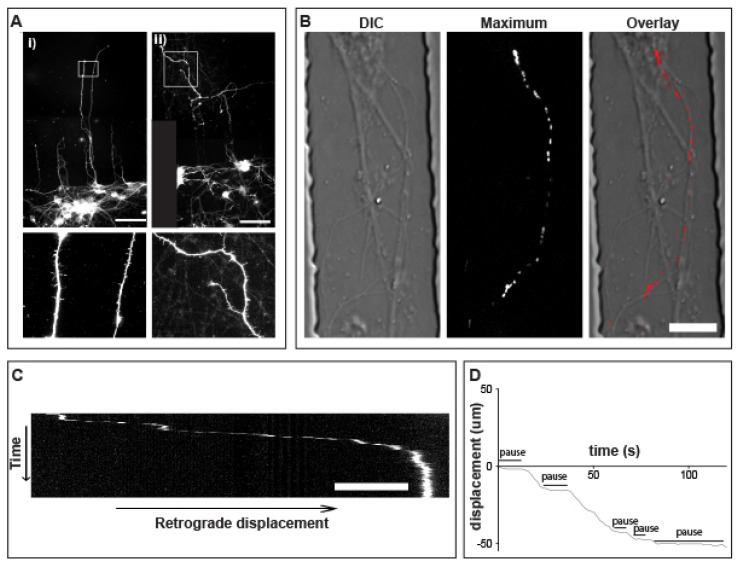
Prolonged pauses dominate the QD-BDNF transport in MSN dendrites. (**A**). (i) Map 2 shows immunofluorescence of dendrites in striatal neuron culture crossing short microgrooves. Bottom inset from (i) highlights dendritic spines. (ii) DARPP-32 immunofluorescence, showing medium spiny neurons in culture. Bottom inset from (ii) shows MSN dendritic spines stained with DARPP-32. (**B**). High-power (100×) images of dendrites in the short microgrooves. Differential interference contrast (DIC), maximum projection of QD-BDNF retrograde transport and color overlay from Movie 2. (**C**). Kymograph of QD-BDNF dendrite transport from Movie 2. (**D**) displacement vs. time plot of QD-BDNF dendrite transport from Movie 2. Scale bars = 20 μm (**A**), 5 μm (**B**,**C**).

After 5–7 days in culture (DIV 5–7), dendrites of striatal neurons were observed crossing the short microgrooves into the distal chamber (Figure 2E). MSN dendrite crossing was further confirmed by immunostaining with MAP2 and DARPP-32 (Figure 4A). To follow single-molecule transport, fluidic isolation was established to prevent diffusion to the cell body chamber and QD-625 labeled mono-biotinylated BDNF (QD-BDNF) was added to the distal dendrites (central chamber, Figure 2A). After 1 h, transport was examined in the proximal dendrites. All data acquired were finally projected to co-align the DIC image to support as a striatal dendrite transport event (Figure 4B). Dendritic transport displays unique features that distinguish it from axonal transport profiles. The most obvious feature of dendritic transport were the presence of very long pauses and the absence of uninterrupted, long-range directional transport typically observed in axons (Figure 4C,D; Movie 2). Dendritic endosomes move in a characteristically slow and often oscillating or staggering trajectory, while also maintaining a clear retrograde vector. The short and oscillatory appearance for dendritic endosome transport is likely a product of the underlying mixed polarity microtubule network. Thus, like what has been observed in axons, dendritic transport maintains a high retrograde directional fidelity. The overwhelming majority of endosomes observed in WT MSN dendrites move in the retrograde direction. These features demonstrate that retrograde dendritic endosomes, like retrograde axonal cargoes, were guided by an undetermined signal that governs directional fidelity over long-range transport, even as they navigate an environment of mixed polarity microtubules.

### 3.6. Retrograde QD-BDNF Transport Is Detained in the Q140 Striatal Dendrites

**Figure 5:**
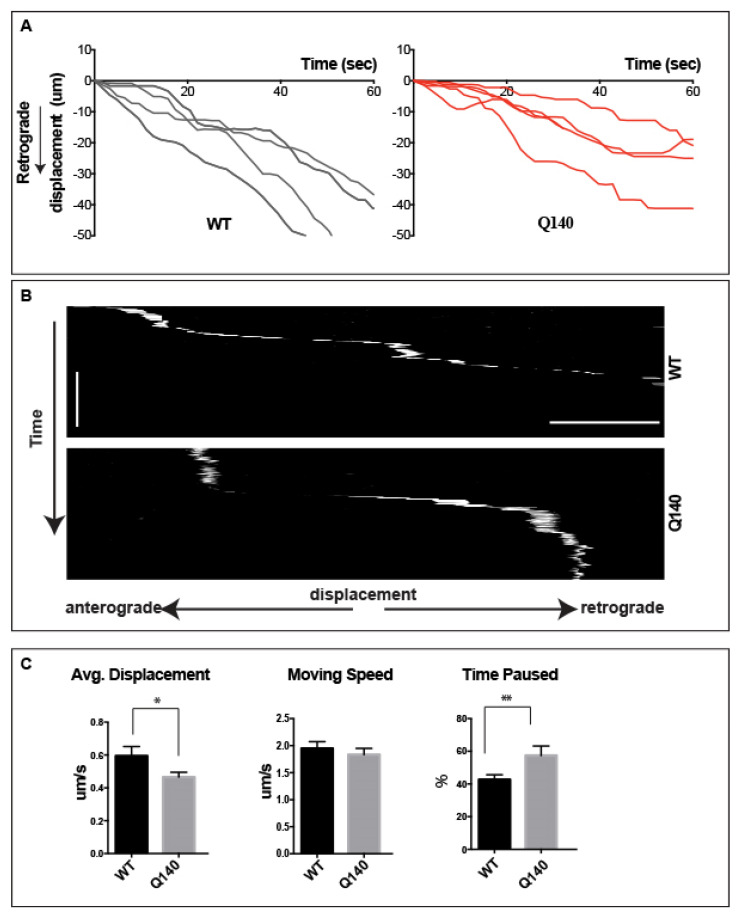
Increased pause time of QD-BDNF endosome in Q140 striatal dendrites. (**A**). Displacement vs. time plots of QD-BDNF retrograde transport in dendrites of WT (gray) and Q140 (red) MSNs. 4 from 50 samples represent. (**B**). Kymographs detailing single-molecule QD labeled endosome dendritic transport. (**C**). Bar graphs quantifying averaged displacement, moving speed and percentage of time paused for QD-BDNF in WT and Q140 MSN dendrites. Data were from three individual repeats. Vertical Scale bar = 1 min., horizontal scale bar = 5 μm, * *p* < 0.01, ** *p* < 0.05.

Having established the transport in dendrites, we next set out to examine and compare the retrograde transport of QD-BDNF in wild-type (WT) and homozygous HTT (Q140) MSN dendrites. For QD-BDNF, the average velocity in WT dendrites is 0.59 ± 0.1 µm/s, and in Q140 dendrites average velocity was reduced to 0.46 ± 0.1 µm/s (Figure 5 and Movie 3). Though already quite slow, the average velocities for QD-BDNF containing endosomes in Q140 MSN dendrites was significantly (*p* < 0.01) reduced as compared to WT. To gain further insight towards the reduced average displacement observed in Q140 dendrites we calculated the moving speed of QD-BDNF by measuring the velocity of labeled endosomes during periods of active transport, eliminating time spent paused. This measurement addresses whether reductions in average displacement result from a general slowing of retrograde transport or from other factors, including excessive pausing or changes in direction. Measurement of moving speed reveals no significant change in QD-BDNF containing endosomes in WT dendrites (1.94 ± 0.2 µm/s) as compared to Q140 (1.79 ± 0.1 µm/s; Figure 5B). To better understand the mechanism(s) underlying the average displacement and moving speed data, we next measured the percentage of time spent paused. Measurement of percentage of time paused reveals that endosomes spend a nearly equal proportion of their time paused as they do in motion. For QD-BDNF, percent time paused was significantly (*p* < 0.05) increased in Q140 dendrites as compared to WT (49.4 ± 7%, WT/QD-BDNF vs. 60.8 ± 0.5%, Q140/QD-BDNF Figure 5C). 

## 4. Discussion

One of the hallmarks of HD is the impaired neurotrophic support of BDNF from the cortex to striatum, resulting in striatal degeneration [64]. It has been previously stated that the physiological effects of BDNF are primarily mediated by its high-affinity tropomyosin-related kinase receptor (TrkB), leading to the activation of the downstream MAPK, PI3K and PLCγ pathways [65,66]. Activation of these pathway aids in neurogenesis, gliogenesis, neurite outgrowth, and enhanced neuronal survival. Thus, it was important to validate the effects of BDNF on the downstream signaling pathways such as the MAPK/ERK and PI3K-PKB/AKT signaling pathways. Several studies have shown that altered BDNF/TrkB signaling has detrimental effects on neuronal survival, differentiation, and synaptogenesis. Our results show a reduced level of pAkt and pCREB in the Q140 cells (Figure 1A), which is increased when induced with BDNF for 30 min. Previously reported mutated huntingtin suppresses the expression of several receptors through an inhibition of CREB binding, and the *BDNF* gene is one of the CREB-targets [67,68,69,70]. This implies that a spatiotemporal supply of BDNF is critical for prompt responses from downstream signaling molecules. 

Microfluidic devices offer significant advantages providing new insights into co-culture and compartmentalization studies for neuronal functions, restoring neuronal issues and neurodegeneration [71,72,73]. The design flexibility and high-precision control of neuronal connectivity have helped in providing solutions to many methodological challenges such as controlling microenvironments of dendrites, synapse formation and regulation and single-molecule axonal transport [74,75]. To further understand the cause behind the BDNF deficiency in Q140, we have attempted to establish a physiologically relevant study of BDNF transport along the axon and dendrite in a model of Huntington’s disease. We traced the ligand/receptor endosome transport of purified recombinant BDNF conjugated with quantum dots (QD-BDNF) [76,77] in the primary neurons, from a Q140 mouse model, co-cultured in a microfluidic chamber. For this study, we designed a custom microfluidic neuronal culture device for the dual purpose of isolating cortical soma from distal axons and for isolating striatal soma from distal dendrites [60]. In this way, we established a co-culture system that can examine the key transport elements of the cortical–striatal circuit (Figure 2). Previous studies have found significant decrease in BDNF release and altered transport of BDNF vesicles in the zQ175 mice (a knock-in model of HD) cortical neurons and their projections to co-cultured striatal neurons [16,78]. Our current work supports these data and advances the field by evaluating single-molecule transportation of BDNF along the cortical–striatal axis without protein overexpression. Quantum dot labeled purified BDNF was used to study axonal anterograde transport in cortical neurons from the medium chamber (NeoCortex) to the central synapse chamber and dendritic retrograde transport in striatal neurons from the central synapse chamber to the striatal cell chamber in the co-cultured microfluidic chamber (Figure 2).

When observing the anterograde transport in cortical axons, the first important finding was the equal distribution of QD-BDNF endosomes for both anterograde and retrograde directions. This differs from retrograde transport, where all endosomes move exclusively in one direction [79]. We furthermore observe that endosomal transport direction was maintained over long distances. However, direction was occasionally reversible and again sustained throughout distance (Figure 3). This finding demonstrates that endosomes in axons generally move in a single direction over long distances but maintain the ability to change direction through an unknown mechanism. Although there are reports of Huntingtin’s role in controlling vesicular directionality in neurons, its direct involvement in changing transport direction is still not clear [80]. To examine anterograde transport only, retrograde-moving endosomes were eliminated for further analysis. Average anterograde displacement of QD-BDNF was significantly reduced (*p* < 0.01) in Q140 cortical axons as compared to WT (Figure 3A). This indicates that a general transport defect is present in Q140 cortical axons, impeding anterograde transport. Though a trend toward reduced speed was measured, this did not reach statistical significance (Figure 3C). Previous studies have reported that mHTT inhibits axonal transport via interaction with kinesin or cytoplasmic dynein subunits [81]. In our study, the initial instant velocity in Q140 cortical axons is altered in comparison to the WT neurons, implying that kinesin’s instant driving force may not be responsible for the observed changes. Percent time paused was significantly increased (*p* < 0.01) for QD-BDNF in the Q140 background versus WT (Figure 3C), indicating that mHTT or other undetermined factors may interfere with kinesin’s activity, causing the increased pausing. Thus, increased pause time is responsible for the decreased averaged displacement in Q140 axons. 

Our findings demonstrate that dendrite retrograde transport proceeds similarly to axon transport when ligands were added to fluidically isolated distal dendrites. Transport in dendrites can be distinguished from axons, however, by the presence of long pauses accounting for approximately an equal amount of time as active transport. Thus, it is suggested that dendrite retrograde transport differs vastly from axon retrograde transport which is characterized by long distance transits interrupted by brief pauses (Figure 4). The finding of processive retrograde transport in dendrites is somewhat surprising, considering the mixed polarity of microtubules present in the dendritic compartment. This observation suggests that endosomes must either switch motors from dynein to kinesin to maintain retrograde direction, or selectively transit between microtubules that are polarized in the proper orientation to maintain a retrograde direction with a single motor system or employ a combination of these strategies. This microtubule orientation change and motor switching may underpin the observations behind the exaggerated pausing in addition to the endosomes that switch direction over very short distances. It appears that within dendrites, endosome direction is being governed by an unidentified “driver” signal that coordinates with the motor protein activity, the selectivity of transport, and a subset of correctly polarized microtubules. Given this complex environment, the observation of lengthy pauses in dendrites is reasonable when compared to axons, which have a single polarity for microtubule arrays. Further work is warranted to identify the factors that govern cargo directional control in dendritic compartments.

Keeping in consideration the retrograde transport observed in dendrites, we compared the retrograde transport of QD-BDNF in wild and homozygous HTT. We observed that endosomal transport of QD-BDNF proceeds exclusively in the retrograde direction in both WT and Q140 striatal neurons. As compared to WT, the average displacement of retrograde QD-BDNF-labeled endosomes in Q140 striatal dendrites is significantly reduced (*p* < 0.01), while time spent paused is significantly increased (*p* < 0.05). Measurements of moving speed showed no change in genotype or cargo, suggesting that motor proteins are operating normally (Figure 5). In conclusion, the data demonstrates that BDNF’s transport in Q140 striatal dendrites is slower or delayed as compared to WT, mediated by increased time spent paused, and that this behavior ultimately influences the downstream signaling pathways.

Quantum dot labelling offers several advantages over organic fluorophores, commonly used for in vivo or in vitro labeling [82]. However, the disadvantages of quantum dots shall be acknowledged. The cadmium and selenium ion core of quantum dots, which is further polymerized via mercaptoacetic acid or PEG-silica, are cytotoxic. Moreover, quantum dots under light stimulation emit high to low noise blinking signals which trap electrons energy and can be phototoxic to cells. Since neuron are very sensitive, our study uses the quantum dot labelling technique to observe transport for short periods of time [76,83]. Additionally, results were drawn based on a comparative analysis of quantum dots in wild-type and quantum dots in mutant neuronal cultures. In addition, the microfluidic co-culture system enables us to establish an in vitro cortical–striatal axis that directly mirrors the relevant in vivo architecture. We considering the system to be compatible with live cell imaging, intracellular trafficking of signaling molecules in healthy and in diseased primary neurons. Additionally, we view it to help to understand the mechanisms at work, as here for the transport deficiency in HD, and provide a fundamental culture system that can be easily adapted to evaluate potential therapeutics [84]. Although the in vitro reconstitution simplifies the cortical–striatal neuronal circuit, it cannot completely replace the in vivo system in terms of the complexity during the whole brain’s development and the circuit’s formation.

Despite these limitations, our study finds a dysregulation in the transport direction, governed by QD-BDNF transport in Q140 cortical–striatal axis. In WT cortical axons, QD-BDNF showed fewer anterograde moving endosomes, while in Q140 cortical axons a greater percentage of QD-BDNF endosomes were observed moving in the anterograde direction. On the contrary, in striatal dendrites of Q140, we observe a slower and reduced percentage of QD-BDNF endosomes moving in the retrograde direction. In addition, we also come across extended pausing of QD-BDNF in Q140 as compared to WT. Taken together, BDNF transport in Q140 along cortical–striatal axis is globally impeded. In summary, these data indicate that neurotrophic transport is uniquely susceptible to changes occurring in the mutant Huntington background, and that these changes have influence over the signaling that regulates long distance BDNF transport along the entire cortical (axon) to striatal (dendrite) axis.

## 5. Conclusions

Taken together, the analysis supports the hypothesis that an alteration in the transport of BDNF contributes to MSN degeneration in HD. This report expands the hypothesis by demonstrating that the spatiotemporal supply of QD-BDNF is disrupted along the cortical (axon) to striatal (dendrite) axis in HD model neurons. While the exact mechanism(s) underlying these finding remain undetermined, HTT should be considered a key suspect in mediating the BDNF-specific defect. Future studies will benefit from uncovering signaling pathways underlying delinquent transport along axons and dendrites in HD. While additional mechanisms undoubtedly contribute to neurodegeneration in HD, spatiotemporal supplies of BDNF remains central as initiating and precipitating factors. Our study describes a fundamental cell culture system that possesses utility for advancing the study of axonal transport and trophic signaling in healthy and in diseased primary neurons, thus providing a fundamental culture system that can be easily adapted to evaluate potential therapeutics for neurodegenerative diseases.

## Figures and Tables

**Table 1:** List of all antibodies used along with their catalog number.

Reagents (Antibodies)	Source	Cat#	Used in Manuscript
Primary antibodies			
Rabbit anti-p44/42 ERK1/2 (137FS)	Cell Signaling Technology, Inc., Danvers, MA, USA	4695S	Figure S3 (WB, 1:1000)
Rabbit anti-phospho-p44/42 ERK1/2 (T202&Y204)	Cell Signaling Technology, Inc., Danvers, MA, USA	4370S	Figure S3 (WB, 1:1000)
Rabbit anti-Akt (11E7)	Cell Signaling Technology, Inc., Danvers, MA, USA	4685S	Figure 1 and Figure S3 (WB, 1:1000)
Rabbit anti-phospho-Akt (S473) (D9E)	Cell Signaling Technology, Inc., Danvers, MA, USA	4060S	Figure 1 and Figure S3 (WB, 1:1000)
Mouse anti-CREB (86B10)	Cell Signaling Technology, Inc., Danvers, MA, USA	9104S	Figure 1 (WB, 1:1000, IF, 1:100)
Rabbit anti-phospho-CREB(S133)	Cell Signaling Technology, Inc., Danvers, MA, USA	9198S	Figure 1 (WB, 1:1000, IF, 1:100)
Rabbit anti-TrkB	EMD Millipore Corp, Burlington, MA, USA	07-225	Figure 1 (WB, 1:1000) and Figure S3 (IF, 1:100)
Rabbit anti-phospho-TrkB(Y490)	Thermo Fisher Scientific, Waltham, MA, USA.	CHDI900520	Figure 1 (WB, 1:1000)
Mouse anti-β-Tubulin iso3 (TuJ1)	AbCam, Cambridge, UK	Ab78078	Figure 2 (IF, 1:250)
Rabbit anti- β-Tubulin iso3 (TuJ1)	AbCam, Cambridge, UK	Ab18027	Figure 1 (WB, 1:1000–2000)
Rabbit anti-Darpp32 (19A3)	Cell Signaling Technology, Inc., Danvers, MA, USA	2306S	Figure 2 and Figure 3 (IF, 1:250)
Mouse anti-huntingtin	EMD Millipore Corp, Burlington, MA, USA	MABN2540	Figure 1 (WB, 1:1000)
Rabbit anti-Tau	Agilent. Santa Clara, CA, USA	A0024	Figure S2 (WB, 1:5000)
Mouse anti-BDNF (N-20)	Santa Cruz Bio, Santa Cruz, CA, USA	SC-546	Figure S2 (WB, 1:1000)
Rabbit anti-MAP2	Covance, Princeton, NJ, USA	PBB-547C	Figure 3 (IF, 1:250)
Rabbit anti-Biotin (D5A7)	Cell Signaling Technology, Inc., Danvers, MA, USA	5597S	Sup.Figure 2 (WB, 1:1000)
Mouse anti-beta Actin (C4)	Santa Cruz Bio, Santa Cruz, CA, USA	SC-47778	Figure S3 (WB, 1:1000)
Alexa Flur488-alpha Tubulin Mono Antibody (DM1A)	eBioscience, San Diego, CA, USA	53-4502-82	Figure 1 (IF, 1:100)
Secondary Antibodies			
HRP-Conjugated Goat anti-Rabbit	Jackson ImmunoResearch, West Grove, PA, USA	111-035-144	For WB (1:10,000)
HRP-Conjugated Goat anti-Mouse	Jackson ImmunoResearch, West Grove, PA, USA	111-035-146	For WB (1:10,000)
Alexa Flur488-conjugated Goat anti-Mouse	Jackson ImmunoResearch, West Grove, PA, USA	115-545-003	For IF (1:1000)
Alexa Flur488-conjugated Goat anti-Rabbit	Jackson ImmunoResearch, West Grove, PA, USA	115-545-062	For IF (1:1000)
Alexa Flur594-conjugated Goat anti-Rabbit	Jackson ImmunoResearch, West Grove, PA, USA	115-585-062	For IF (1:1000)
Alexa Flur647-conjugated Goat anti-Mouse	Jackson ImmunoResearch, West Grove, PA, USA	115-605-003	For IF (1:1000)

## Data Availability

Not applicable.

## Supplementary Materials

The following supporting information can be downloaded at: https://www.mdpi.com/article/10.3390/biology12020157/s1, Figure S1: Scheme of QD-BDNF design. Scheme to show the experimental design. The Quantum-dot 625-streptavidin requires conjugation with the biotin protein as the live imaging tag. In turn, the biotin protein requires BirA ligase attachment to a unique 15 amino acid Avi-tag sequence which must be present on the BDNF protein. In chronological order, 1. construct individual BDNF-Avi proteins, 2. biotinylate BDNF-Avi by BirA and purify the biotinylated proteins, 3. test the biological functionality of biotinylated BDNF (defined as the proteins’ capability of being taken into cells), 4. conjugate quantum-dot (Q-Dot) to the biotinylated BDNF and fabricate the microfluidic chamber, and 5. conduct the live transport in neuron cultured in the microfluidic chamber system. Figure S2: Biotinylated BDNF design, construct, and purification. (**A**). Biotin-BDNF was purified from medium of transfected 293T (proBDNF-biotin with BirA) via Ni-NTA resin. The eluted Biotin-BDNF was marked with “*” sign. (**B**). Biotin-BDNF was WB confirmed with BDNF and biotin antibodies. (**C**). Biotin-BDNF in the elution was quantified based on the concentration known recombinant BDNF (rBDNF). The final concentration in elution is 2 ng/uL. Figure S3: Biotinylated BDNF has biological function. (**A**). Biotin-BDNF (btBDNF) is function on TrkB signaling pathway. The 3T3 stable expressing TrkB cell line was used for btBDNF function study. Akt and Erk42/44 under the TrkB signaling pathway was phosphorylated after 30 min incubation with 25 ng/mL Biotin-BDNF (btBDNF). No reaction was detected under the TrkA pathway (on the TrkA stable express 3T3 cells). Recombinant BDNF (rBDNF) was taken as positive control. (**B**). The endosomal pattern was also observed with the staining after Biotin-BDNF’s incubation, effect was same as rBDNF’s treatment. Elution buffer gave the negative result. (**C**). After conjugation to the Q-Dot, the transport was detected with QD-BDNF in the 3T3(TrkB) cell line. Figure S4: Bidirectional transport of QD-BDNF in cortical axons. Displacement vs. time plots of QD-BDNF anterograde/retrograde transport in axons of WT (gray) cortical neurons (**A**). Kymographs detailing single-molecule axon transport. (**B**) Bi-directional transport of QD-BDNF in WT cortical axon, (**a**) one retrograde transport event and (**b**) one anterograde transport event. Vertical Scale bar = 1 min, horizontal scale bar = 5 μm. Figure S5: the original images of western blots for figure 1; Figure S6: the original images of western blots for figure 2. Movie 1: Perturbed anterograde transport of QD-BDNF in Q140 cortical axons. Live imaging of QD-BDNF containing endosomes anterogradely transported in cortical axon. The anterograde transport was from soma body (up) to neurite terminal (proximal, bottom). Q140 cortical axon is identified slower transport and increased pausing when comparation to WT neurons. The data were presented in Figure 2. Movie 2: Prolonged pauses dominate QD-BDNF transport in MSN dendrites. Live imaging of QD-BDNF containing endosomes in striatal dendrite. The retrograde transport was from neurite terminal (proximal, bottom) to soma body (up). The data were presented in Figure 3. Movie 3: Increased pause time of QD-BDNF endosome in Q140 striatal Dendrites. Live imaging of QD-BDNF containing endosomes retrogradely transported in striatal dendrite. The retrograde transport was from neurite terminal (proximal, bottom) to soma body (up). Q140 striatal dendrite are characterized with slower transport and increased pausing when compared to WT neurons. The data were presented in Figure 4.
