# Supplementary material for: Failure to Thrive: Impaired BDNF Transport along the Cortical–Striatal Axis in Mouse Q140 Neurons of Huntington’s Disease
Source: Biology (Basel). 2023 Jan 19;12(2):157. doi: 10.3390/biology12020157 (PMC9952218; doi:10.3390/biology12020157)
Supplement: Supplementary file 1 [file biology-12-00157-s001.zip › biology-2041919-Figure S6.pdf]

Original Figures: WB (4-8-2018)

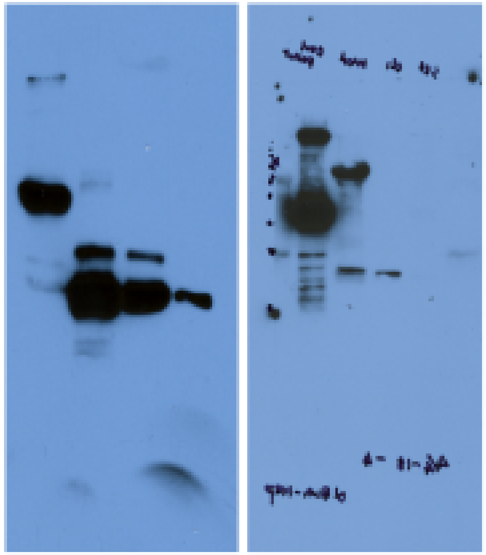

For Sup Fig2B (anti BDNF and Biotin)

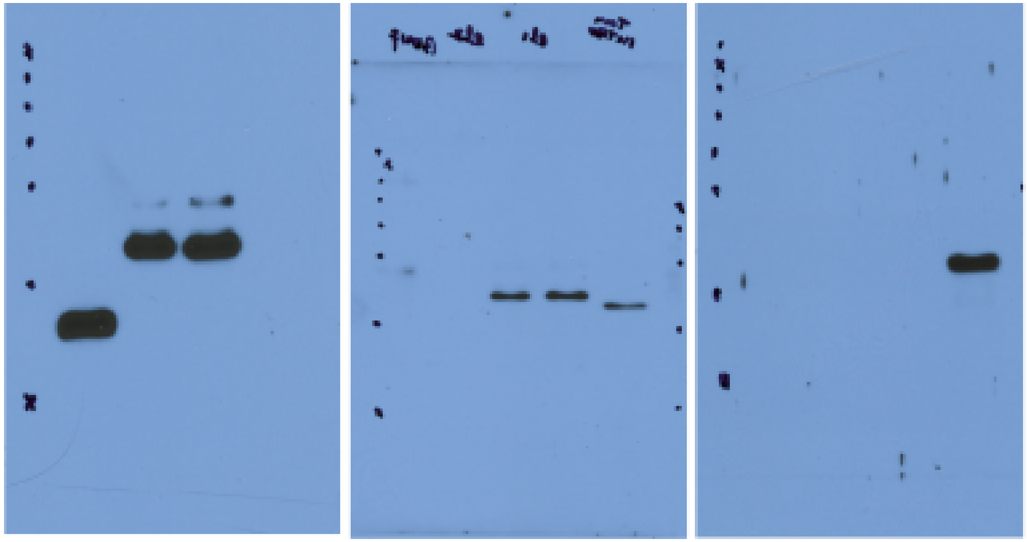

For Sup Fig2C (anti BDNF,Biotin and Tau)

Original Figures: WB (5-8-2018)

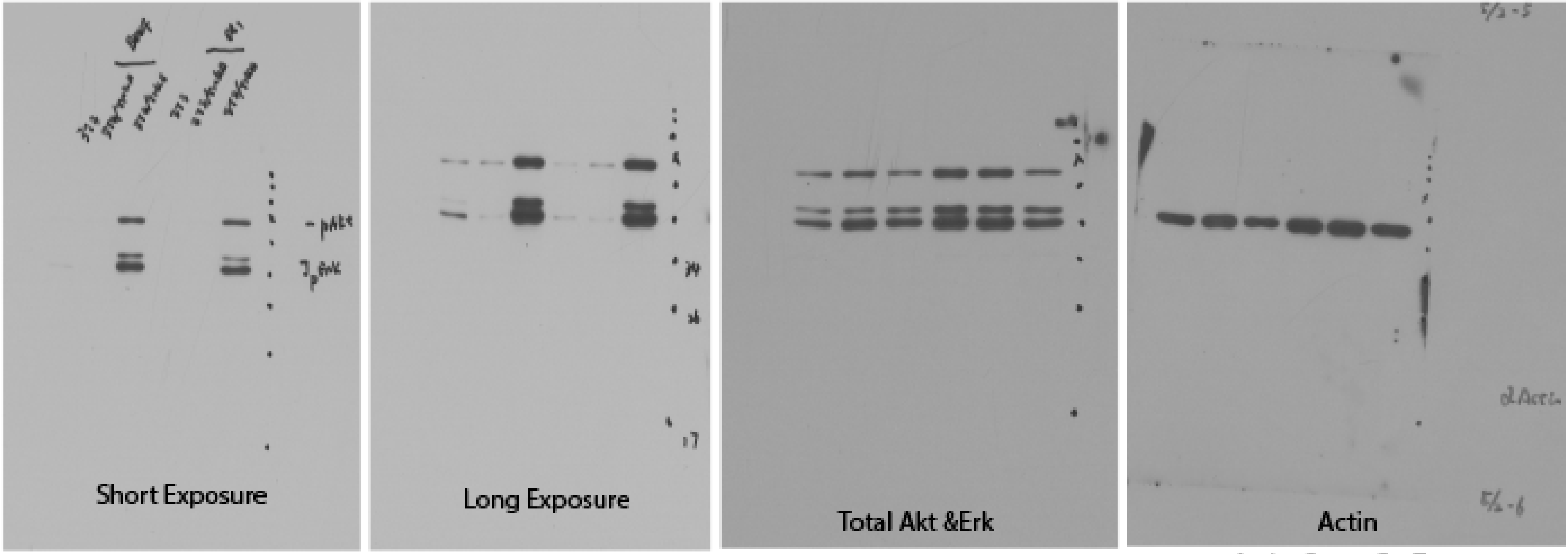

Phos.Akt&Erk (CST, 1:1000)

Akt&Erk (CST, 1:1000)

Actin (Santa CruZ, 1:1000)
