# Supplementary material for: Failure to Thrive: Impaired BDNF Transport along the Cortical–Striatal Axis in Mouse Q140 Neurons of Huntington’s Disease
Source: Biology (Basel). 2023 Jan 19;12(2):157. doi: 10.3390/biology12020157 (PMC9952218; doi:10.3390/biology12020157)
Supplement: Supplementary file 1 [file biology-12-00157-s001.zip › biology-2041919-Firgure S1-Figure S4.pdf]

Supplementary Figures:

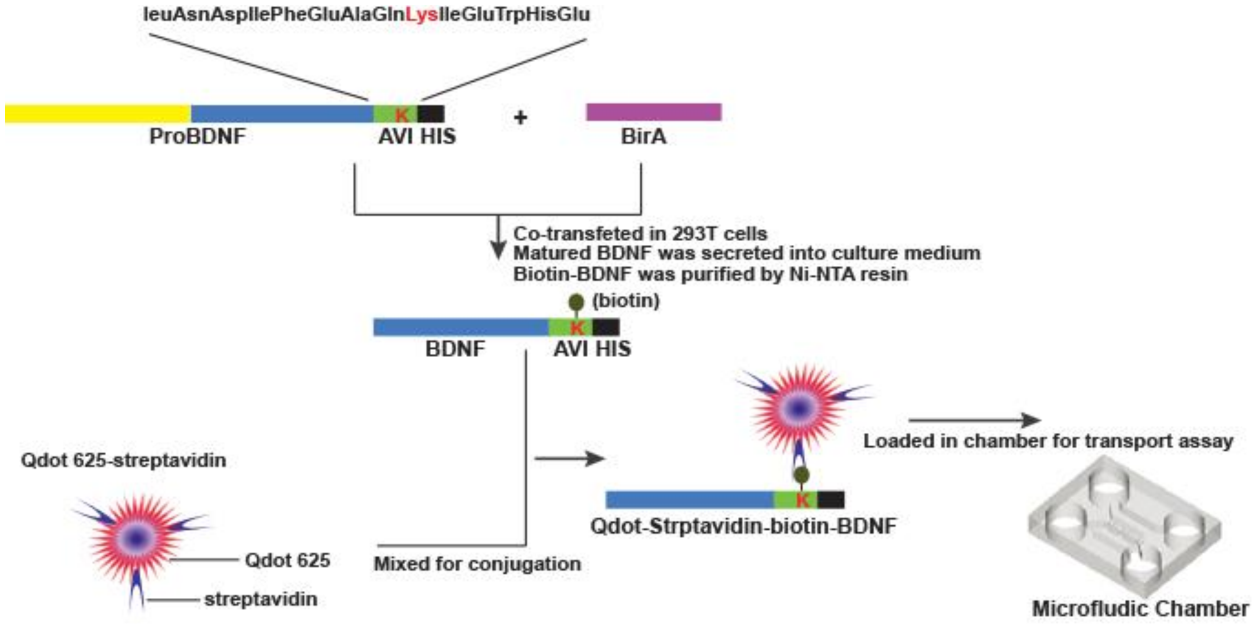

Figure S1. Scheme of QD-BDNF design.

Scheme to show the experimental design. The Quantum-dot 625-streptavidin requires conjugation with the biotin protein as the live imaging tag. In turn, the biotin protein requires BirA ligase attachment to a unique 15 amino acid Avi-tag sequence which must be present on the BDNF protein. In chronological order, 1. construct individual BDNF-Avi proteins, 2. biotinylate BDNF-Avi by BirA and purify the biotinylated proteins, 3. test the biological functionality of biotinylated BDNF (defined as the proteins' capability of being taken into cells), 4. conjugate quantum-dot (Q-Dot) to the biotinylated BDNF and fabricate the microfluidic chamber, and 5. conduct the live transport in neuron cultured in the microfluidic chamber system.

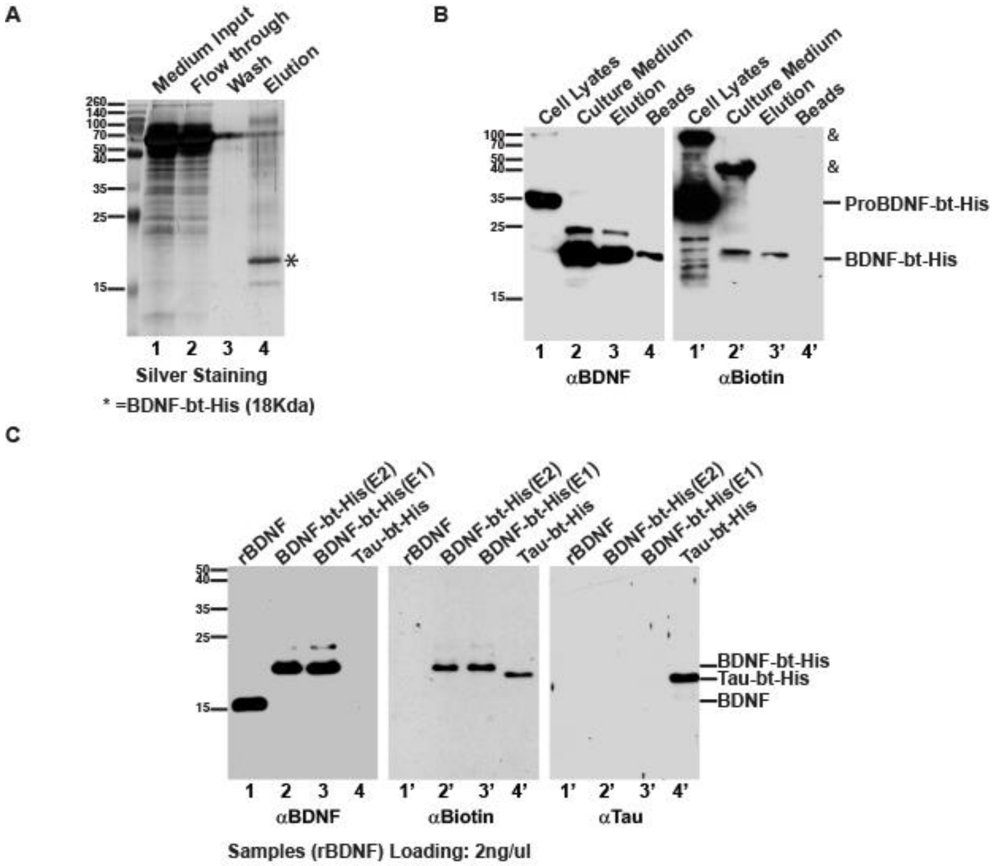

Figure S2. Biotinylated BDNF design, construct, and purification. (A). Biotin-BDNF was purified from medium of transfected 293T (proBDNF-biotin with BirA) via Ni-NTA resin. The eluted Biotin-BDNF was marked with "*" sign. **(B)**. Biotin-BDNF was WB confirmed with BDNF and biotin antibodies. **(C).** Biotin-BDNF in the elution was quantified based on the concentration known recombinant BDNF (rBDNF). The final concentration in elution is 2 ng/uL.

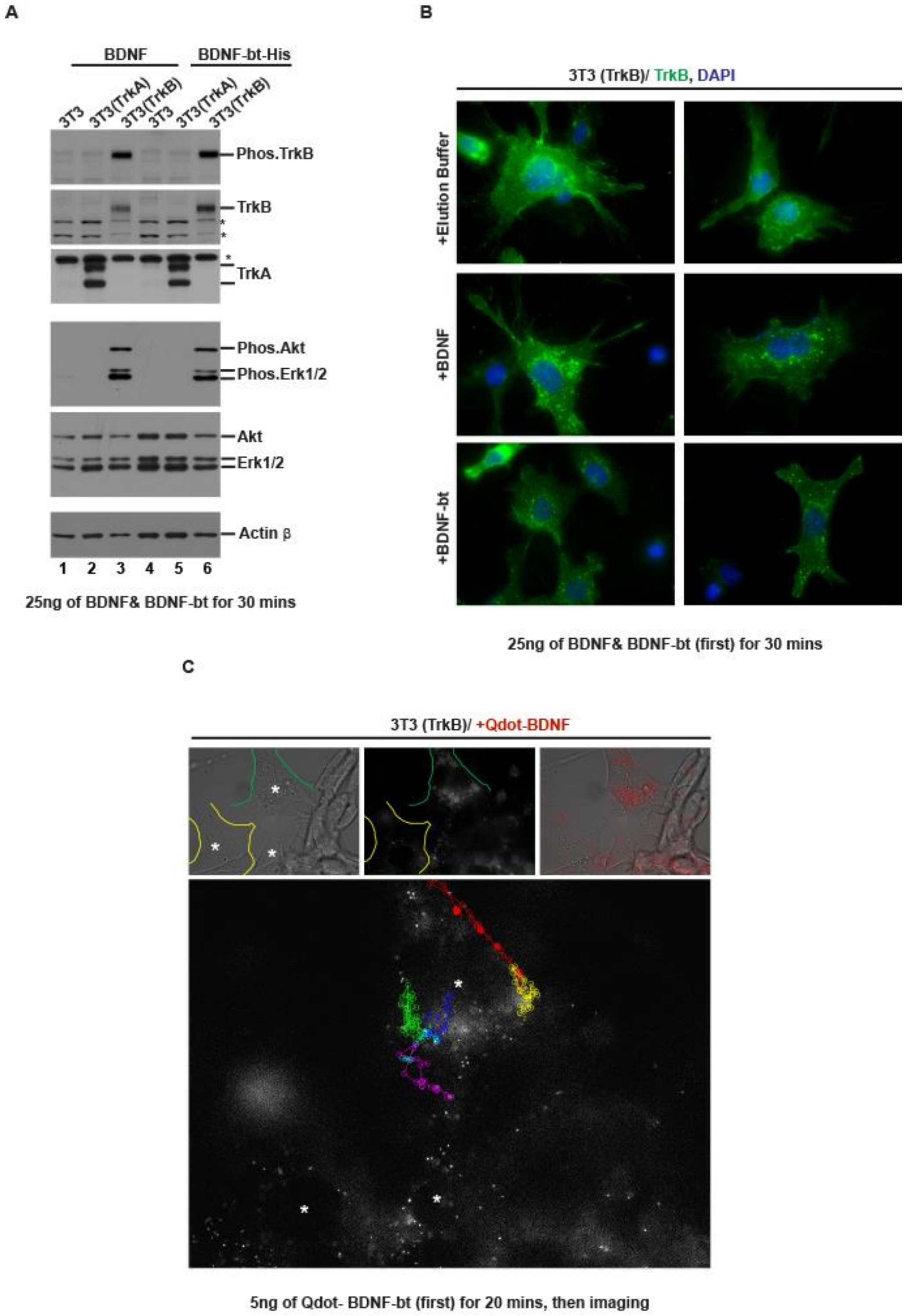

Figure S3. Biotinylated BDNF has biological function. **(A).** Biotin-BDNF (bt-BDNF) is function on TrkB signaling pathway. The 3T3 stable expressing TrkB cell line was used for bt-BDNF function study. Akt and Erk42/44 under the TrkB signaling pathway was phosphorylated after 30 minutes incubation with 25 ng Biotin-BDNF (bt-BDNF). No reaction was detected under the TrkA pathway (on the TrkA stable express 3T3 cells). Recombinant BDNF (rBDNF) was taken as positive control. **(B).** The endosomal pattern was also observed with the staining after Biotin-BDNF's incubation, effect was same as rBDNF's treatment. Elution buffer gave the negative result. **(C).** After conjugation to the Q-Dot, the transport was detected with QD-BDNF in the 3T3(TrkB) cell line.

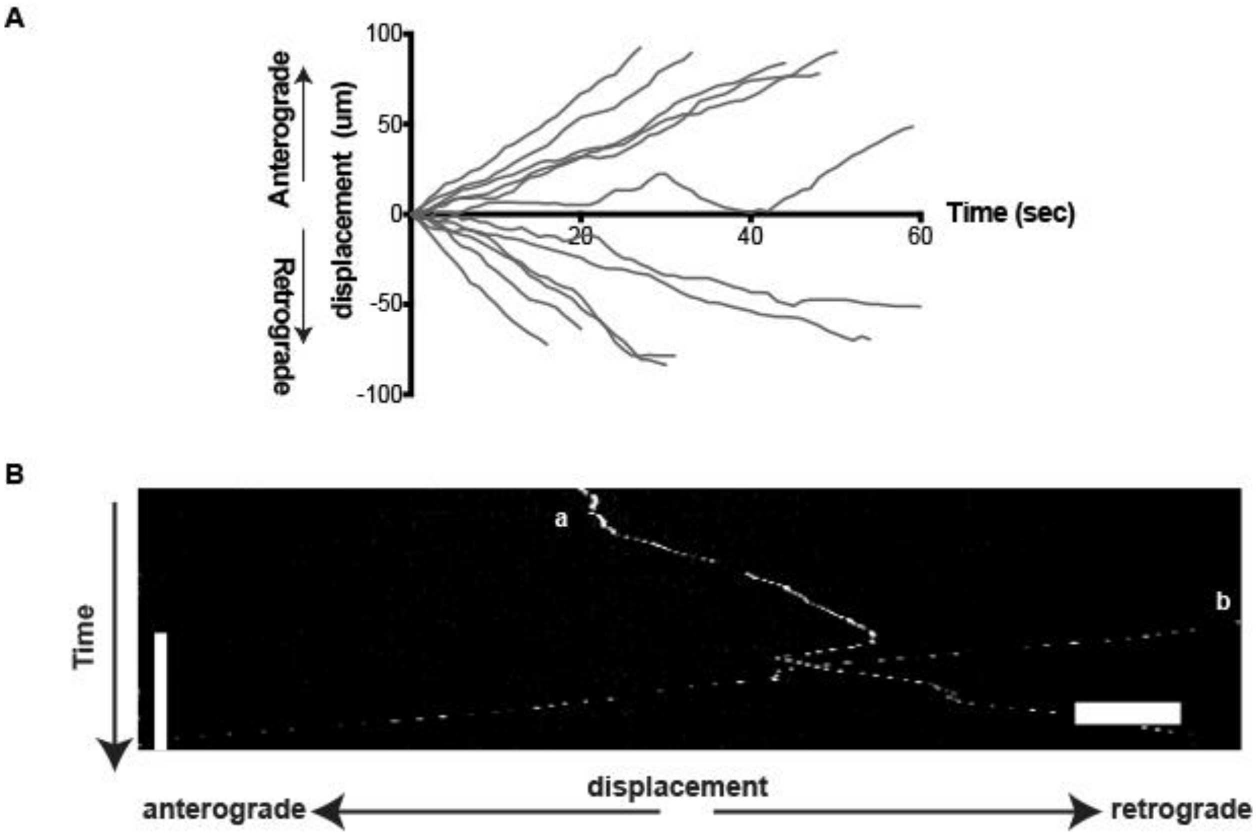

Figure S4. Bidirectional transport of QD-BDNF in cortical axons when added in cortical soma body.

Displacement vs. time plots of QD-BDNF anterograde/retrograde transport in axons of WT (gray) cortical neurons **(A).** Kymographs detailing single molecule axon transport. **(B)** Bi-directional transport of QD-BDNF in WT cortical axon, (a) one retrograde transport event and (b) one anterograde transport event. Vertical Scale bar = 1 min., horizontal scale bar = 5 μm
