# Supplementary figures and images for: Failure to Thrive: Impaired BDNF Transport along the Cortical–Striatal Axis in Mouse Q140 Neurons of Huntington’s Disease
Source: Biology (Basel). 2023 Jan 19;12(2):157. doi: 10.3390/biology12020157 (PMC9952218; doi:10.3390/biology12020157)

Figure S5: Original Figures: WB for Figure 1

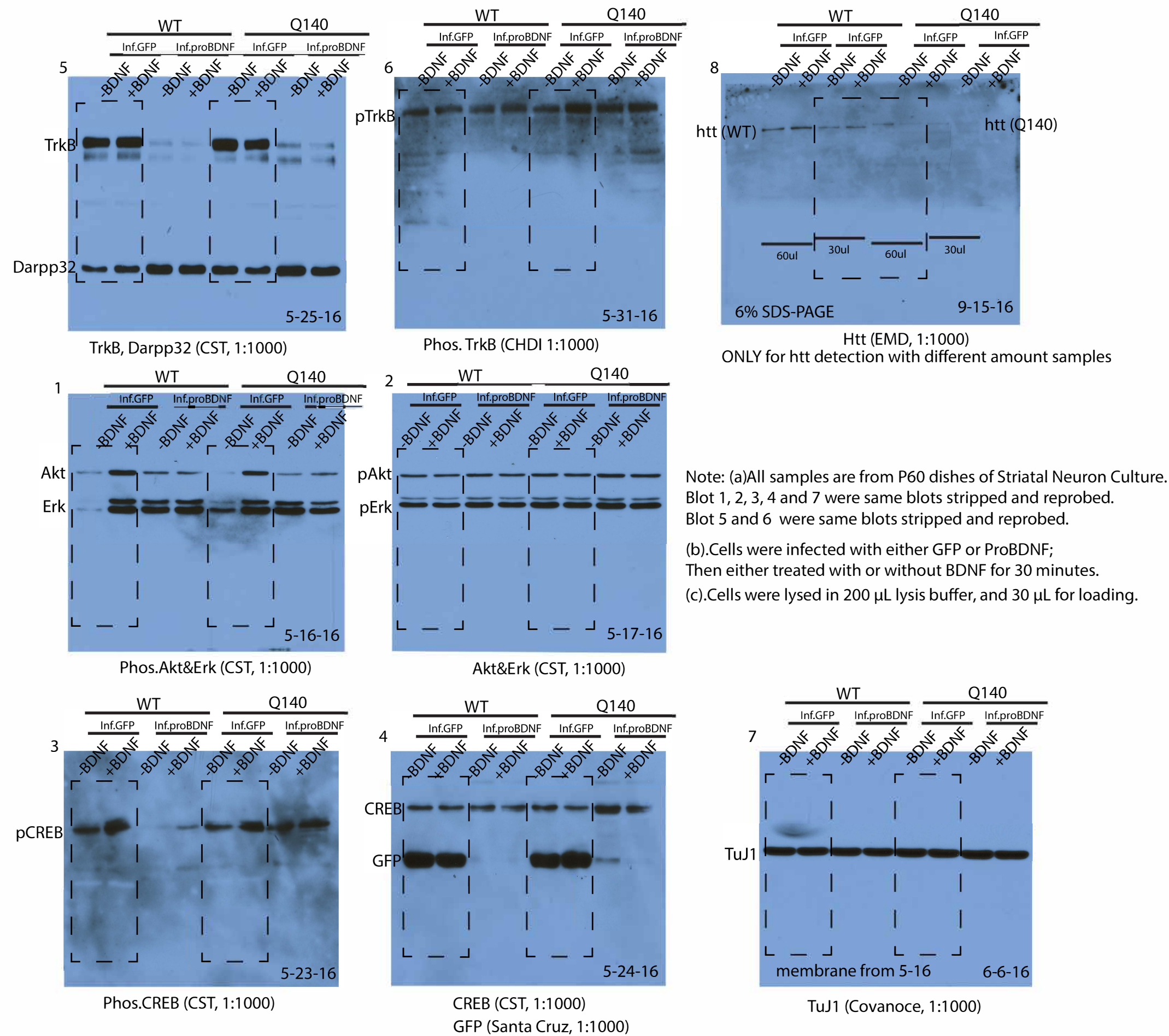

Supplement: Supplementary file 1 [file biology-12-00157-s001.zip › biology-2041919-Figure S5.pdf]
